# Role of 4-Thiazolidinone–Pyrazoline/Indoline Hybrids Les-4369 and Les-3467 in BJ and A549 Cell Lines

**DOI:** 10.3390/cells13121007

**Published:** 2024-06-08

**Authors:** Karolina Kosińska, Bartosz Skóra, Serhii Holota, Yulia Shepeta, Anna Tabęcka-Łonczyńska, Roman Lesyk, Konrad A. Szychowski

**Affiliations:** 1 Department of Biotechnology and Cell Biology, Medical College, University of Information Technology and Management in Rzeszow, Sucharskiego 2, 35-225 Rzeszow, Poland; bskora@wsiz.edu.pl (B.S.); atabecka@wsiz.edu.pl (A.T.-Ł.); rlesyk@wsiz.edu.pl (R.L.); kszychowski@wsiz.edu.pl (K.A.S.); 2 Department of Pharmaceutical, Organic and Bioorganic Chemistry, Danylo Halytsky Lviv National Medical University, Pekarska 69, 79010 Lviv, Ukraine; golota_serg@yahoo.com; 3 Department of Organic Chemistry and Pharmacy, Lesya Ukrainka Volyn National University, Volya Avenue 13, 43025 Lutsk, Ukraine; 4 Department of Pharmaceutical Chemistry, National Pirogov Memorial Medical University, Pirogov 56, 21018 Vinnytsia, Ukraine; shepeta.yulia@gmail.com

**Keywords:** 4-thiazolidinone derivatives, cancer, ROS, LDH, caspase-3 activity, PPARγ

## Abstract

Cancer is one of the most important problems of modern societies. Recently, studies have reported the anticancer properties of rosiglitazone related to its ability to bind peroxisome proliferator receptor γ (PPARγ), which has various effects on cancer and can inhibit cell proliferation. In this study, we investigated the effect of new 4-thiazolidinone (4-TZD) hybrids Les-4369 and Les-3467 and their effect on reactive oxygen species (ROS) production, metabolic activity, lactate dehydrogenase (LDH) release, caspase-3 activity, and gene and protein expression in human foreskin fibroblast (BJ) cells and lung adenocarcinoma (A549) cells. The ROS production and caspase-3 activity were mainly increased in the micromolar concentrations of the studied compounds in both cell lines. Les-3467 and Les-4369 increased the mRNA expression of *PPARG*, *P53* (tumor protein P53), and *ATM* (ATM serine/threonine kinase) in the BJ cells, while the mRNA expression of these genes (except *PPARG*) was mainly decreased in the A549 cells treated with both of the tested compounds. Our results indicate a decrease in the protein expression of AhR, PPARγ, and PARP-1 in the BJ cells exposed to 1 µM Les-3467 and Les-4369. In the A549 cells, the protein expression of AhR, PPARγ, and PARP-1 increased in the treatment with 1 µM Les-3467 and Les-4369. We have also shown the PPARγ modulatory properties of Les-3467 and Les-4369. However, both compounds prove weak anticancer properties evidenced by their action at high concentrations and non-selective effects against BJ and A549 cells.

## 1. Introduction

Anticancer drugs that are highly effective without dangerous side effects are still in demand. One of the most common cancer diagnoses is lung cancer, causing 18% of deaths in the world [1]. In 2023, 1,958,310 new cancer cases and 609,820 deaths, with 238,340 new lung cancer cases and 127,070 deaths related to lung cancer, were reported in the USA [2]. The causes of lung cancer include smoking, occupational exposure, alcohol consumption, radon, air pollution, dietary factors, genetic factors, and psychological factors [3].

Due to their biological activity, heterocyclic compounds like 4-thiazolidinone (4-TZDs) are interesting given their use in medicine [4]. The most popular and most widely used derivatives of the above-mentioned compounds include 2,4-thiazolidinedione, 2-thioxo-4-thiazolidinone, 2-alkyl(aryl)-substituted, and 2-R-amino(imino)-substituted 4-TZDs [5]. 4-TZD derivatives have anti-inflammatory, antiproliferative, antidiabetic, and antitumor mechanisms of action, which make them an interesting area for research [4]. In addition, 4-TZDs can be modified to obtain a wide range of potentially active compounds whose properties may be interesting for medical applications [6]. For instance, troglitazone and ciglitazone showed the ability to induce changes in the Ca^2+^ influx in rat liver epithelial cells GN4 [7,8,9]. In turn, ciglitazone induced reactive oxygen species (ROS) overproduction in mouse preadipocyte cell line 1B8 [10], myoblasts C2C12 [11], and rat liver epithelial cells GN4 [7,8,9]. Other TZD derivatives, i.e., STG28 (troglitazone derivative) and OSU-CG12 (ciglitazone derivative), induced endoplasmic reticulum (ER) stress in prostate cancer cells (LNCaP) [12]. The mechanism of the anticancer-based action of 4-TZDs is focused mainly on cancer-related biotargets, e.g., JNK simulating phosphatase-1 (JSP-1) protein, tumor necrosis factor α (TNFα), and/or anti-apoptotic bio-complex (Bcl-XL-BH3) [4]. Moreover, increased production of ROS after treatment with 4-TZDs was noted in various cancer cell lines [13,14,15,16,17], which can be regarded as another anticancer-based factor of these compounds [14]. As demonstrated in the reports, rosiglitazone, troglitazone, and pioglitazone, which are modulators of peroxisome proliferator receptor gamma (PPARγ), are representatives of heterocycles [5]. In the case of lung cancer, PPARγ has antiproliferative and proapoptotic properties and may also inhibit the development of primary tumors. Furthermore, as shown by Reddy et al., PPARγ agonists may prevent cancer cell metastasis by inhibiting the acquisition of the migratory capacity of migrating cells [18]. In turn, PPARγ can also regulate inflammation and immunity responses and, as a result of oxidation stress (OS), can regulate the expression of antioxidant enzymes like superoxide dismutase (SOD) and catalase (CAT) [19,20]. In turn, one of the OS-related genes is the ATM serine/threonine kinase (*ATM*) gene encoding a protein responsible for the induction of apoptosis as a result of DNA damage [21] and tumor protein 53 (*P53*), which involves cell apoptosis to eliminate cells with damaged DNA [22]. An important protein in cell apoptosis is poly [ADP-ribose] polymerase 1 (PARP-1), whose activation is a consequence of DNA damage and activation of apoptosis cascades involving caspase-3 and caspase-7, which finally lead to cell death due to DNA cleavage [23,24,25].

Aryl hydrocarbon receptor (AhR) dimerizes with a nuclear translocator (ARNT), leading to the binding and regulating xenobiotic-responsive elements (XREs) (DNA-specific region in the nucleus) [26]. The complex AhR-ARNT induces the transcription of *CYP1A1*, *CYP1A2*, and *CYP1B1* genes, which are engaged in the phase I metabolism of xenobiotics [27,28,29]. Moreover, the AhR-ARNT complex can regulate genes encoding proteins involved in the phase II metabolism of NAD(P)H quinone dehydrogenase (NQO1), glutathione S-transferase A2 (GSTA2), UDP-glucuronosyltransferase 1A1 (UGT1A1), and UDP-glucuronosyltransferase 1-6 (UGT1A6) [27,28]. As a result of the action of exogenous chemicals, AhR may activate nicotinamide adenine dinucleotide phosphate (NADPH) oxidase to produce ROS, which leads to damage to the vascular endothelium [30]. Interestingly, many papers have shown the crosstalk between PPARγ and the AhR as a response to specific xenobiotics in many cell models [31,32,33,34]. As a result of OS, the PPARγ-AhR crosstalk may regulate the expression of CAT and SOD in cells [19,35,36]. Last but not least, the impact of the new 4-TZD-based molecules on PPARγ and their possible agonist properties have been shown in different human cell lines, such as normal fibroblast (BJ), squamous cell carcinoma (SCC-15), lung carcinoma (A549), colon adenocarcinoma (CACO-2), and neuroblastoma (SH-SY5Y) cells [13,37,38,39]. The new 4-TZD derivatives should be investigated given the promising anticancer properties of these compounds presented in the available literature [13,14,37,39].

**Figure 1:**
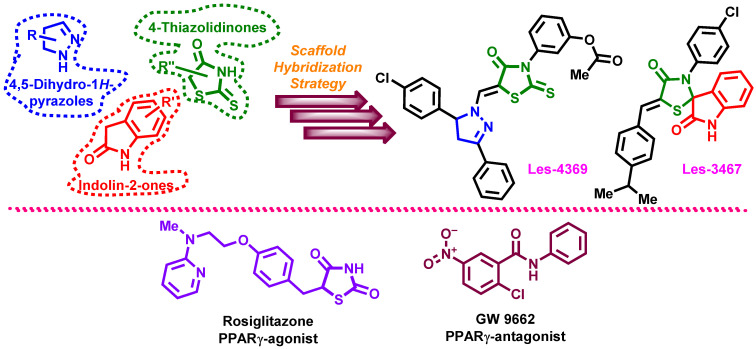
Applied design strategy for 4-thiazolidinone hybrids Les-4369 and Les-3467. Structures of the potential PPARγ-agonist rosiglitazone and PPARγ-antagonist GW9662 used in the experiments.

Among the different chemotypes of 4-thiazolidinone hybrids, pyrazoline- and indoline-bearing molecules draw attention as candidates to be used for the design of potential multi-target anticancer agents and in-depth studies of their pharmacological properties [6,15,39,40]. Therefore, this study aimed to investigate the new 4-TZD-based derivatives (Z)-5-[5-(4-chlorophenyl)-3-phenyl-4,5-dihydropyrazol-1-ylmethylene]-3-(3-acetoxyphenyl)-2-thioxothiazolidin-4-ones (Les-4369) and (5′Z)-3′-(4-chlorophenyl)-5′-[(4-isopropylphenyl)methylene]spiro[indoline-3,2′-thiazolidine]-2,4′-dione (Les-3467) (Figure 1) in terms of their anticancer effects and potential PPARγ modulatory properties.

We examined the impact of 1, 10, 50, and 100 nM and 1, 10, 50, and 100 μM concentrations of Les-4369 and Les-3467 on the cellular functions of BJ and A549 cells. For this purpose, we examined the metabolic activity, lactate dehydrogenase (LDH) release, ROS production, and activity of caspase-3 in BJ and A549. In addition, we investigated the mRNA expressions of genes such as *PPARG*, *P53*, and *ATM* and the levels of PPARγ, AhR, ARNT, and PARP-1 protein expression.

## 2. Materials and Methods

### 2.1. Reagents

Dulbecco’s Modified Eagle’s Medium (DMEM) and Ham’s F-12K (Kaighn’s) medium (F12K) were purchased from Corning (Manassas, VA, USA). Fetal bovine serum (FBS), Universal RNA Purification Kit, Fast Probe qPCR Master Mix, radioimmunoprecipitation buffer (RIPA), and Perfect^TM^ Tricolor Protein Ladder were purchased from EURx (Gdańsk, Poland). Phosphate-buffered saline (PBS) without Ca^2+^ and Mg^2+^, rosiglitazone, trypsin, penicillin, streptomycin, sodium pyruvate, Tris-HCl, hydrocortisone, sodium bicarbonate, 2′,7′-dichlorodihydrofluorescein diacetate (H_2_DCF-DA), iodonitrotetrazolium chloride (INT), beta-nicotinotinamide adenine dinucleotide sodium salt (NAD), methoxyphenazine methosulfate (MPMS), 2-chloro-5-nitrobenzanilide (GW9662), dimethyl sulfoxide (DMSO), 4-(2-hydroxyethyl)-1-piperazineethanesulfonic acid (HEPES), 3-((3-cholamidopropyl)dimethylammonio)-1-propanesulfonate (CHAPS), ethylenediaminetetraacetic acid (EDTA), dithiothreitol (DTT), Ac-DEVD-pNA (caspase-3 substrate), Calcein–AM, bisbenzimide trihydrochloride (Hoechst 33342), and resazurin sodium salt were purchased from Sigma-Aldrich (St. Louis, MO, USA). The High-Capacity cDNA—Reverse Transcription Kit and TaqMan^®^ probes corresponding to specific genes encoding *ACTB* (Hs01060665_g1), *PPARG* (Hs00234592_m1), *P53* (Hs01034249_m1), and *ATM* (Hs00175892_m1) and HRP-conjugated anti-mouse (31430) and anti-rabbit (31460) secondary antibodies were obtained from ThermoFisher (Forest City, CA, USA). The mouse primary anti-AhR antibodies (67785-1-Ig) were kindly gifted by Proteintech (Manchester, UK). Other mouse primary anti-glyceraldehyde 3-phosphate dehydrogenase (GAPDH, AC033) antibodies and rabbit anti-PPARγ (A11183), anti-ARNT (A19532), and anti-PARP-1 (A19596) antibodies were purchased from ABClonal (Düsseldorf, Germany). The PVDF membrane with a pore size of 0.45 μm was obtained from Santa Cruz Biotechnology (Dallas, TX, USA). Bovine serum albumin (BSA) was purchased from Glentham Life Sciences (Crosham, UK).

The synthesis and physicochemical data of the tested compounds were described previously in [40] for Les-4369 and in [41] for Les-3467. All stock solutions were prepared by dissolving the compounds in DMSO. The final concentration of DMSO in the culture medium was always 0.1%.

### 2.2. Cell Culture

Human lung adenocarcinoma cells (CCL-185, A549) and human foreskin fibroblasts (CRL-2522, BJ) were obtained from the American Type Culture Collection (ATCC, distributor: LGC Standards, Łomianki, Poland). The A549 cell line was maintained in the F12K medium with 10% of FBS and 0.1% antibiotic (penicillin and streptomycin). The BJ cell line was maintained in the DMEM medium with 10% of FBS, 0.1% antibiotic, and 4 mm L-glutamine. The cells were maintained in an incubator at 37 °C in a humidified atmosphere with 5% CO_2_. For ROS measurements, resazurin reduction, and caspase-3 activity assays, the cells were seeded in 96-well culture plates at a density of 4 × 10^3^ (for 24 and 48 h treatment) per well and initially cultured 24 h before the experiment. The cells for qPCR were seeded in 12-well culture plates at a density of 1.2 × 10^5^ per well for 24 h. For protein measurement, the cell lines were seeded onto 6-well plates at a density of 1.83 × 10^5^ per well and initially cultured for 24 h. For fluorescence-based microscopic observation, the cells were seeded in a ⌀35 mm culture dish at the density of 1.2 × 10^5^ cells per culture dish for 24 h treatment.

### 2.3. Intracellular ROS Level

Briefly, the BJ and A549 cells were incubated with 5 µM of H_2_DCFDA for 30 min in serum-free and phenol-red-free medium. After that, the cells were incubated with fresh medium with increasing concentrations (1, 10, 50, and 100 nM and 1, 10, 50, and 100 μM) of Les-3467 or Les-4369 for 24 and 48 h. The fluorescence intensity was measured by a microplate reader (FilterMax F5) at the maximum excitation and emission wavelengths of 535 and 595 nm, respectively.

### 2.4. Resazurin-Reduction-Based Cell Viability Assay

Briefly, 100 µM of resazurin sodium salt was prepared with 1% of FBS and culture medium. After 24 h and 48 h of incubation of the cells with increasing concentrations of Les-3467 and Les-4369, the culture medium was changed to a fresh one containing resazurin (100 µL/well). The plates were incubated at 37 °C. The fluorescence was measured after 30 min and 1 h using a microplate reader (FilterMax F5) at the maximum excitation and emission wavelengths of 535 and 595 nm, respectively.

### 2.5. LDH Cytotoxicity Assay

After treatment of the cells with 1, 10, 50, or 100 nM or 1, 10, 50, or 100 μM of Les-3467 and Les-4369, the culture medium was collected and transferred to a new plate. The analysis of LDH release was performed according to Kaja et al. [42]. In total, 50 µL of buffer A (4 mM INT) and buffer B (6.4 mM NAD and 320 mM lithium lactate with 50 mM MPMS in 200 mM Tris-HCl) was applied to the cell medium and incubated at 30 min in the dark. The absorbance was measured with a microplate reader (FilterMax F5) at the maximum excitation wavelength of 450 nm.

### 2.6. Caspase-3 Activity Assay

The analysis of the caspase-3 activity was performed as in Nicholson et al. (1995) [43]. Ninety-six-well plates with BJ and A549 cells were frozen at −80 °C after 24 h and 48 h treatment with 1, 10, 50, and 100 nM and 1, 10, 50, and 100 μM of Les-3467 and Les-4369 and thawed on the day of experiments. At first, lysis buffer (50 mM HEPES, pH 7.4, 100 mM NaCl, 0.1% CHAPS, 1 mM EDTA, 10% glycerol, and 10 mM DTT) was prepared, and then, the cells were lysed at 10 °C for 10 min. The lysates were incubated with the caspase-3 substrate Ac-DEVD-pNA at 37 °C. The absorbance of the lysates was measured at 405 nm using a FilterMax F5 Multi-Mode microplate reader and continuously monitored for 120 min.

### 2.7. Real-Time PCR Analysis of PPARγ, ATM, and P53 mRNA Expression

The mRNA was isolated with the use of the Universal RNA Purification Kit according to the manufacturer’s instructions after 24 h cell treatment with 1 μM of Les-3467 or Les-4369. To check the RNA quantity, the spectrophotometric method with the maximum excitation and emission wavelengths of 260 and 280 nm was employed with the use of NanoDrop (ND/1000 UV/Vis; Thermo Fisher, Waltham, MA, USA). The amount of RNA was normalized to 300 ng RNA for the BJ cells and 800 ng RNA for the A549 cells. The reverse transcription reaction (RT) was conducted using the obtained RNA and the High-Capacity cDNA Reverse Transcription Kit. The reaction was performed with the use of Fast Probe qPCR Master Mix (2x), Taq-Man probe, and primers for a total volume of 20 µL. The qPCR procedure was executed with the following parameters: 2 min at 50 °C and 10 min at 95 °C, followed by another 40 cycles of 15 s at 95 °C and 1 min at 60 °C. Data analysis was performed with the ΔΔCt method, while the threshold value (Ct) for the samples was established during the exponential phase. The method was validated using the reference β-actin (*ACTB*) gene.

### 2.8. Hoechst 33342- and Calcein–AM-Based Staining

The BJ and A549 cells were exposed to 1 μM of Les-3467 and Les-4369, and the cells were cultured for an additional 24 h. After this period, the cells were washed with PBS and exposed to Hoechst 33342 and Calcein–AM diluted in medium without FBS at a final concentration of 10 µM and 4 µM, respectively. The cells were incubated for 10 min in an atmosphere of 5% CO_2_ and 37 °C, washed one time in PBS, and visualized using a fluorescence microscope (LSM 700, ZEISS).

### 2.9. Western Blot

For the Western blot assay, the A549 and BJ cells were seeded on 6-well plates and exposed to 1 µM of Les-4369, 1 µM of Les-3467, 1 µM of GW9662, and 1 µM of rosiglitazone or co-treated with these compounds. After the experiments, the cells were lysed using ice-cold RIPA buffer with protease inhibitors. To determine the protein concentration, the bicinchoninic acid (BCA) method was used [44]. The concentration of each protein sample was normalized, and denaturation with 5× Laemmli buffer (Bio-Rad, Hercules, CA, USA) was carried out for 5 min at 95 °C. The prepared samples were separated by electrophoresis with 7.5% SDS–polyacrylamide gel (Bio-Rad). After electrophoresis, proteins were transferred to a PVDF membrane at 35 V, at 4 °C, overnight. The non-specific side blocking was performed using 1% of BSA in TBST for 1 h; then, the primary antibodies specific against PPARγ (1:1000), AhR (1:2000), ARNT (1:2000), PARP-1 (1:1000), and GAPDH (1:100,000) were added for overnight incubation at 4 °C. Subsequently, the PVDF membrane was washed three times for 10 min in TBST, and secondary anti-mouse (1:2000) and anti-rabbit (1:3000) antibodies were added and incubated for 1 h, RT. After this time, the membranes were washed three times for 10 min using TBST. The results were obtained using a chemiluminescent substrate (ECL) in a C-DiGit^®^ Blot Scanner (LI-COR Biosciences—Lincoln, NE, USA). GAPDH was always used as a loading control after stripping the membranes. Densitometry was carried out using the GelQuantNET.

### 2.10. Statistical Analysis

The data are presented as means ±SD of six independent experiments. Each treatment was repeated six times (*n* = 6) and measured in triplicate for colorimetric (LDH release, caspase-3 activity) and fluorometric (resazurin reduction and ROS production) methods. Each treatment was repeated 12 times (*n* = 12) in qPCR and 3 times (*n* = 3) in Western blot. The results were analyzed and illustrated with GraphPad Prism 8.0.1 (GraphPad Software 8.4.3, Boston, MA, USA). The data were analyzed with one-way analysis of variance (ANOVA) with Tukey’s multiple post hoc comparison test; *** *p* < 0.001, ** *p* < 0.01, and * *p* < 0.05 vs. the control cultures. The statistical analysis of the differences between the analyzed groups was carried out at ### *p* < 0.001.

## 3. Results and Discussion

### 3.1. ROS Production

**Figure 2:**
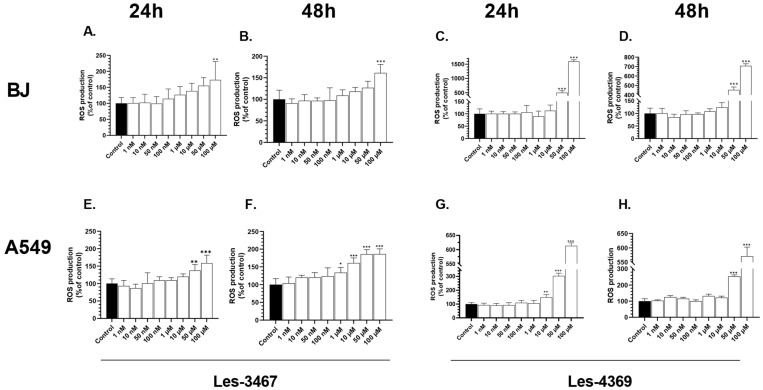
Effects of increasing concentrations of Les-3467 (**A**,**B**,**E**,**F**) and Les-4369 (**C**,**D**,**G**,**H**) (1 nM–100 μM) on the ROS production in the A549 and BJ cell lines after 24 h (**A**,**E**,**C**,**G**) and 48 h (**B**,**F**,**D**,**H**). The statistical significance of each data point was analyzed by Tukey’s test using one-way ANOVA for each study group; * *p* < 0.05, ** *p* < 0.01, and *** *p* < 0.001, compared with control cells.

The first part of our study was focused on investigations of the ROS production caused by Les-4369 and Les-3467, i.e., 4-TZD derivatives, in normal BJ cells and cancer A549 cells. The H_2_DCFDA dye was used to determine the amounts of intercellular ROS. After the 24 h exposure of the BJ cells, only 100 µM Les-3467 caused an increase in ROS production by 73.17%, compared to the control. Moreover, after 48 h, the ROS production increased by 61.32% also in the 100 µM Les-3467 treatment, compared to the control (Figure 2A,B). After 24 h, Les-4369 used at the highest concentrations increased ROS production in the BJ cells. Compared to the control, 50 and 100 µM Les-4369 increased ROS production by 500.36% and 1501.18%, respectively. After 48 h, the ROS production increased significantly at the 50 and 100 µM concentrations by 352.22% and 608.26%, respectively, compared to the control (Figure 2C,D).

After 24 h, the ROS production in the A549 cells increased significantly at the 50 μM and 100 μM Les-3467 concentrations by 37.45% and 58.63%, respectively, compared to the control. After 48 h, the ROS production increased in the 1, 10, 50, and 100 µM concentration variants by 33.93, 60.85, 86.00, and 86.38%, respectively, compared to the control (Figure 2E,F). In the 24 h Les-4369 treatment, the ROS production increased at the 10, 50, and 100 µM concentrations by 48.95, 203.71, and 513.52%, respectively, compared to the control. After 48 h, the ROS production increased in the 50 and 100 µM treatments by 153.99 and 471.48%, respectively, compared to the control (Figure 2G,H).

Our results showed an increase in the ROS production in the BJ cells only at the highest concentrations of Les-4369 and Les-3467 after 24 h and 48 h treatments. However, the A549 cells seem to be more sensitive to the tested compounds in which Les-3467 increases the ROS production in the range of 1 to 100 µM after 48 h treatment, while Les-4369 increases the ROS production in the range of 10 to 100 µM after 24 h treatment (Figure 2).

**Figure 3:**
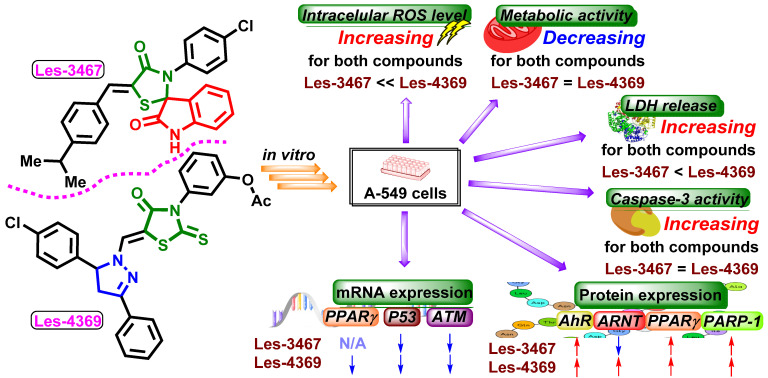
Schematic presentation of the cytotoxic effects and potential mechanisms of action of derivatives Les-3467 and Les-4369. N/A—no effect.

**Figure 4:**
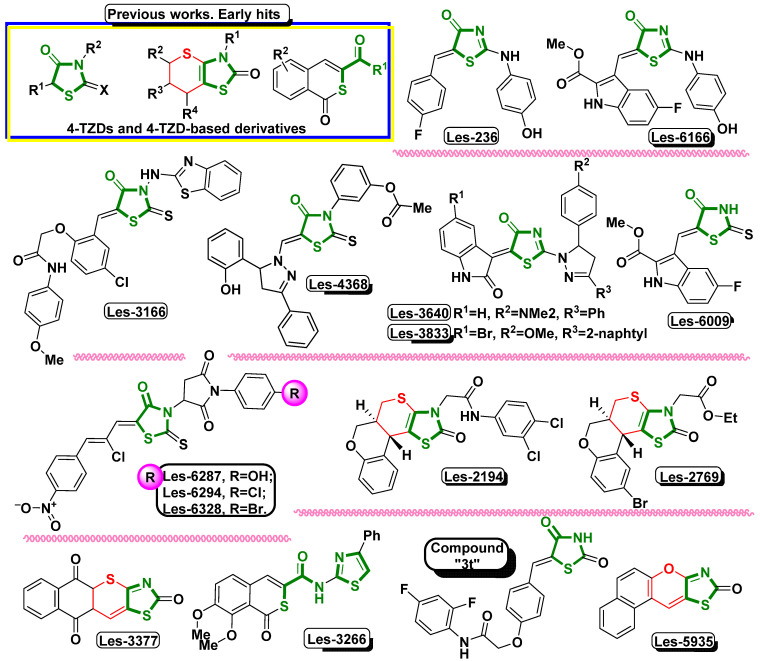
Structures of 4-TZDs and 4-TZD-based derivatives used in the present and previous studies.

These results suggest the ROS-dependent mechanism of action of the tested compounds (Figure 3). Similar results were presented by Szychowski et al. (2017), who showed that 4-TZD-based derivatives Les-2194, Les-3377, and Les-3640 (Figure 4) increased ROS production in the SCC-15 cell line after 24 h [14]. Furthermore, Les-3377, Les-2194, and Les-3640 increased ROS production in the BJ, SCC-15, CACO-2, and A549 cell lines [13]. Another 4-TZD derivative, Les-4368 (Figure 4), induced apoptosis in mammalian leukemia cells via G_0_/G_1_ arrest and ROS production [5]. Interestingly, Les-3377 caused ROS production in mouse embryo fibroblasts (3T3-L1), without significant changes in the case of Les-2194 and Les-3640 [45]. As shown by, e.g., Kaminskyy et al. and Abd Al Moaty et al., the ability to induce ROS overproduction is one of the well-described anticancer strategies, based on which the new TZD-based derivatives are being designed [5,46].

### 3.2. Resazurin Reduction Assay

**Figure 5:**
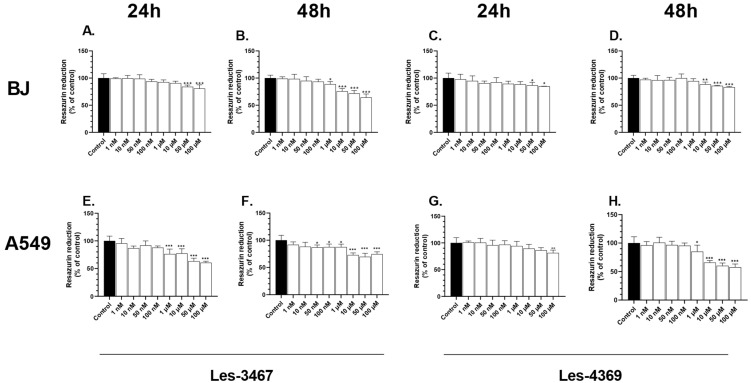
Metabolic activity of increasing concentrations of Les-3467 (**A**,**B**,**E**,**F**) and Les-4369 (**C**,**D**,**G**,**H**) (1 nM–100 μM) in the A549 cancer cell line and the BJ normal cell line after 24 h (**A**,**E**,**C**,**G**) and 48 h (**B**,**F**,**D**,**H**). The statistical significance of each data point was analyzed by Tukey’s test using one-way ANOVA for each study group; * *p* < 0.05, ** *p* < 0.01, and *** *p* < 0.001, compared with control cells.

The reduction of resazurin is used as an indicator of cell metabolism [47]. After the 24 h exposure of the BJ cells to Les-3467, the reduction of resazurin decreased at the 50 µM and 100 µM concentrations by 15.98 and 19.17%, respectively, compared to the control. After 48 h, the effect of decreased resazurin reduction was enhanced, as the 1, 10, 50, and 100 µM concentrations decreased resazurin reduction by 11.14, 21.18, 28.31, and 35.67%, respectively, compared to the control (Figure 5A,B). After 24 h, only the 50 and 100 µM Les-4369 concentrations decreased resazurin reduction by 13.28 and 14.96%, respectively, compared to the control. After 48 h, the resazurin reduction was decreased at the 10, 50, and 100 µM concentrations by 11.57, 14.18, and 16.74%, respectively, compared to the control (Figure 5C,D).

In the A549 cell line, Les-3467 decreased resazurin reduction in the 1, 10, 50, and 100 µM treatments by 23.71, 22.64, 36.73, and 39.49%, respectively, compared to the control. After 48 h, resazurin reduction was decreased by 13.09, 12.40, 12.40, 27.03, 30.28, and 25.48% in variants with 0.05, 0.1, 1, 10, 50, and 100 µM, respectively, compared to the control (Figure 5E,F). After 24 h, only 100 µM Les-4369 decreased resazurin reduction by 18.71%, compared to the control. However, after 48 h, a 14.95, 34.29, 39.76, and 42.44% decrease was observed at the 1, 10, 50, and 100 µM concentrations, respectively, compared to the control (Figure 5G,H).

Many papers indicate that ROS can induce cytotoxic effects in cells by causing redox imbalance [48,49]. The results obtained in this study showed decreased levels of resazurin reduction in the Les-3467 and Les-4369 treatments of the BJ and A549 cells. Moreover, this effect was strengthened in the BJ and A549 cells after the 48 h exposure to both tested compounds. Our results suggest a greater impact of Les-3467 on metabolic activity than Les-4369. As reported by Bar et al. (2022), 4-TZD derivatives, such as Les-2769 and Les-3266 (Figure 4), decreased the metabolic activity in BJ and SCC-15 cells [37]. Skóra et al. (2022) described a decrease in metabolic activity in BJ and A549 cells after 24 and 48 h exposure to Les-3166, Les-5935, Les-6166, and Les-6009 (Figure 4) [39]. In turn, Szychowski et al. (2021) showed a Les-3377-induced decrease in metabolic activity in BJ cells [13]. Another study of Les-3377 showed decreased metabolic activity in 3T3-L1 cells at 2 and 10 μM of the compound [45]. Finiuk et al. (2023) described a decrease in metabolic activity in SCC-15 cells exposed to 4-TZD derivatives Les-6287, Les-6294, and Les-6328 (Figure 4) [50]. Therefore, based on the above-shown results and cited papers, it can be concluded that our findings are in line with the present state of the art concerning 4-TZD derivatives.

### 3.3. LDH Release Assay

**Figure 6:**
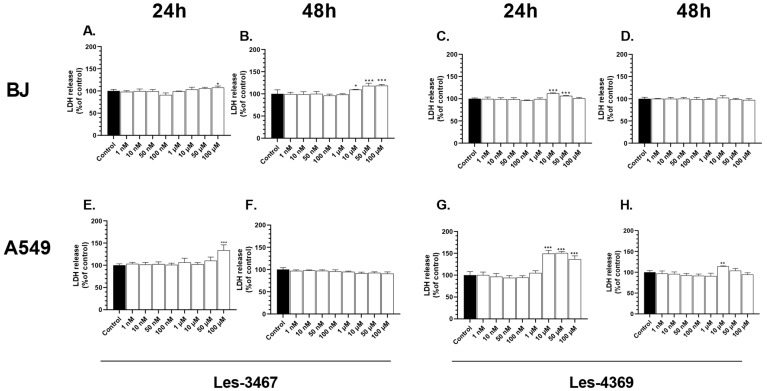
LDH release level at increasing concentrations of Les-3467 (**A**,**B**,**E**,**F**) and Les-4369 (**C**,**D**,**G**,**H**) (1 nM–100 μM) in the A549 cancer cell line and the BJ normal cell line after 24 h (**A**,**E**,**C**,**G**) and 48 h (**B**,**F**,**D**,**H**). The statistical significance of each data point was analyzed by Tukey’s test using one-way ANOVA for each study group; * *p* < 0.05, ** *p* < 0.01, and *** *p* < 0.001, compared with control cells.

LDH is a marker of dead cells. When the integrity of the cell membrane is disturbed, LDH is released into culture medium [51]. After 24 h, only 100 μM Les-3467 increased the LDH release from the BJ cells by 7.78%, compared to the control. After 48 h, increased LDH release was observed at the 10, 50, and 100 μM concentrations by 9.55, 18.16, and 18.63%, respectively, compared to the control (Figure 6A,B). After 24 h, we observed a significant 12.04 and 6.7% increase in the LDH release induced by 10 and 50 μM Les-4369, respectively, compared to the control, whereas no significant changes in the LDH release were observed after 48 h (Figure 6C,D).

After 24 h, the concentration of 100 μM Les-3467 significantly increased the LDH release from the A549 cells by 33.72%, compared to the control. After 48 h, no changes in the LDH release were caused by Les-3467 (Figure 6E,F). After 24 h, Les-4369 applied in the concentration range of 1, 10, 50, and 100 μM induced a 33.93, 60.85, 86.00, and 86.38% increase in the LDH release, respectively, compared to the control. After 48 h, only the concentration of 10 μM of Les-4369 increased the LDH release significantly by 14.35%, compared to the control (Figure 6G,F).

LDH is a marker of cell membrane damage, which may suggest the beginning of apoptosis mechanisms, necrosis, or other forms of damage to cells [51,52]. Our experiment showed increased LDH release in the A549 and BJ cells after 24 h exposure to Les-3467 and Les-4369. In turn, an increase in the LDH release level in high micromolar concentrations of the tested compounds indicates cell death. Similarly, Bar et al. showed that other 4-TZD derivatives, such as Les-2769 and Les-3266, increased LDH release mainly at high concentrations in SCC-15 cells [37]. Finiuk et al. (2023) reported increased LDH release from SCC-15 cells treated with high concentrations of Les-6287, Les-6294, and Les-6328 [50]. Another study conducted by Szychowski et al. (2019) indicated increased LDH release caused by the Les-236 TZD derivative in BJ, SCC-15, and SH-SY5Y cell lines [53]. In contrast, in the present study, no changes in the A549 cells were induced by Les-3467 after 48 h, and similar results were obtained in the BJ cells exposed to Les-4369. The absence of changes in the LDH level in long time intervals may be a result of the high toxicity of the studied compounds followed by LDH decomposition [54]. Moreover, Les-4369 and Les-3467 induced cell damage in the BJ and A549 cells, suggesting the non-selective action of these compounds against both normal and cancer cell lines, which is regarded as a well-described proapoptotic factor.

### 3.4. Caspase-3 Activity Assay and Fluorescence Microscope Analysis

**Figure 7:**
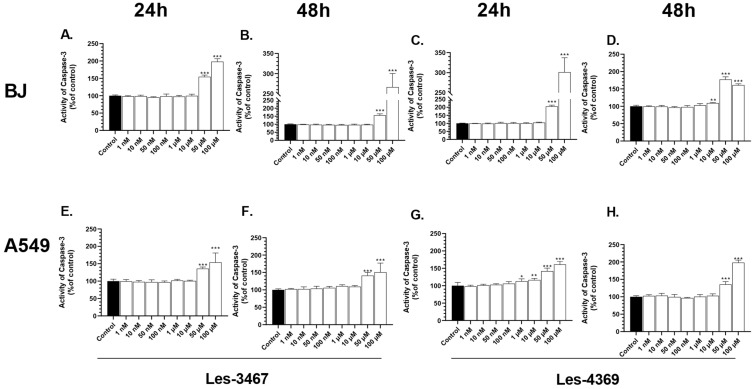
Caspase-3 activity at increasing concentrations of Les-3467 (**A**,**B**,**E**,**F**) and Les-4369 (**C**,**D**,**G**,**H**) (1 nM–100 μM) in the A549 cancer cell line and the BJ normal cell line after 24 h (**A**,**E**,**C**,**G**) and 48 h (**B**,**F**,**D**,**H**). The statistical significance of each data point was analyzed by Tukey’s test using one-way ANOVA for each study group; * *p* < 0.05, ** *p* < 0.01, and *** *p* < 0.001, compared with control cells.

Caspase-3 is a protease with proteolytic functions that lead to programmed cell death via apoptosis [55]. After 24 h, caspase-3 activity in the BJ cells increased significantly at the 50 and 100 μM Les-3467 concentrations by 54.78 and 98.23%, respectively, compared to the control. After 48 h, the caspase-3 activity increased at the same concentrations by 56.29 and 166.42%, respectively, compared to the control (Figure 7A,B). In the Les-4369 treatments, the caspase-3 activity increased significantly by 105.45 and 201.37% at 50 and 100 μM, respectively, compared to the control. After 48 h, the caspase-3 activity increased significantly at 10, 50, and 100 μM by 9.41, 77.32, and 61.16%, respectively, compared to the control (Figure 7C,D).

After 24 h, a 36.03 and 54.00% increase in caspase-3 activity in the A549 cell line was observed at the 50 and 100 μM Les-3467 concentrations, respectively, compared to the control. After 48 h of treatment with the same concentrations, the caspase-3 activity increased by 41.17 and 50.71%, respectively, compared to the control (Figure 7F,G). In the 24 h Les-4369 treatment, the caspase-3 activity increased at 1, 10, 50, and 100 μM by 13.09, 16.54, 42.07, and 61.09%, respectively, compared to the control (Figure 7G). On the other hand, the caspase-3 activity after 48 h increased by 35.56 and 98.31% only at the Les-4369 concentrations of 50 and 100 μM (Figure 7H).

Apoptosis is the most desirable way to remove cancer cells from organisms due to the non-pro-inflammatory character of this process [56]. In addition, caspase-3 regulates the apoptosis processes by cleaving the inhibitor of caspase-activated DNase (ICAD), causes activation of caspase-activated DNase (CAD), and in consequence, induces DNA fragmentation [57,58]. Our results indicate increased activity of caspase-3 in the BJ and A549 cells at high concentrations of Les-3467 and Les-4369. Bar et al. (2022) reported similar results of caspase-3 activity in BJ cells mainly at high concentrations of Les-3266 and Les-2769 (Figure 4) [37]. In the case of Les-3166, Les-6009, and Les-6166, the highest caspase-3 activity in BJ and A549 cells was found at 50 and 100 μM [39]. Szychowski et al. (2021) showed increased caspase-3 activity only at 10 μM Les-3640 in BJ cells, while the highest activity in A549 cells was recorded at 1 and 10 μM [13].

**Figure 8:**
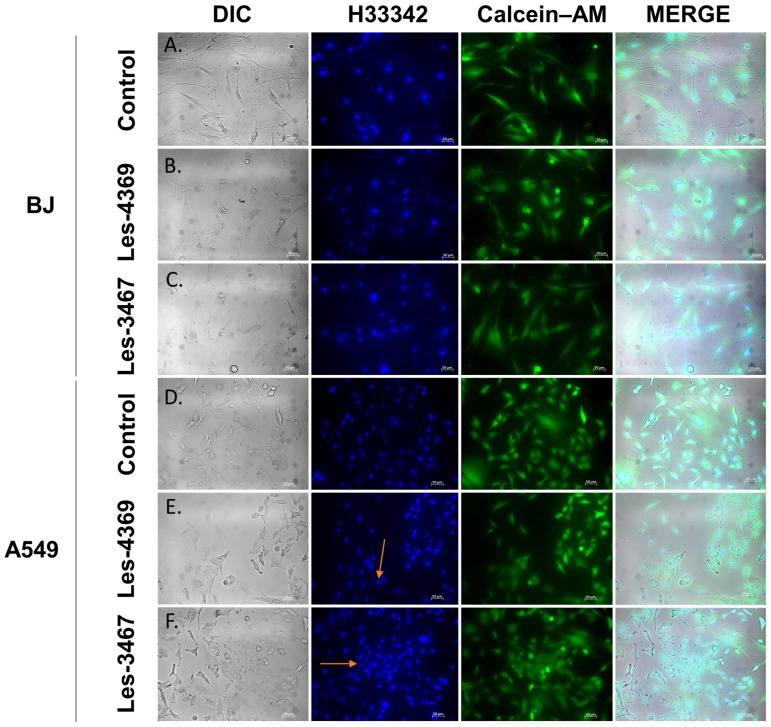
Confocal imaging with Hoechst 33342 (H33342) and Calcein–AM staining of the BJ and A549 line cells after the exposure to 1 μM of Les-4369 (**B**,**E**) and 1 μM of Les-3467 (**C**,**F**) and without the compounds (**A**,**D**) after 24 h treatment. The orange arrows mark apoptotic vesicles. Magnification of 200× was used.

Apoptotic cells exhibit nuclear condensation and DNA fragmentation, which can be detected via vital staining with Hoechst 33342. Hoechst 33342 binds to DNA fragments in apoptotic bodies and emits blue fluorescence, whereas living cells exhibit esterase activity, which is visualized as green fluorescent light via Calcein–AM staining [59]. The BJ and A549 cells were stained with Hoechst 33342 and Calcein–AM to determine the presence of apoptosis and assess cell viability (Figure 8). In the BJ cells, we observed the formation of apoptotic vesicles after the cell treatment with Les-4369, with no changes in cells exposed to Les-3467. The viability of cells was unchanged in both groups, compared to the control. A number of apoptotic vesicles in the A549 cells were observed after the exposure to both studied compounds (1 µM Les-3467 and 1 µM Les-4369) without a reduction in the cell number.

In addition, the fluorescence microscope analysis revealed the formation of apoptotic bodies in the A549 cells exposed to 1 µM of Les-3467 and Les-4369, which indicates the final stage of apoptosis, characterized by membrane blebbing and the formation of protrusions on the cell surface [60]. In summary, our compounds Les-4369 and Les-3467 induced the activation of caspase-3 in both cell lines at high concentrations; this indicates the non-selective action of these compounds, which may prove their weak anticancer properties.

### 3.5. Gene and Protein Expression Analysis

**Figure 9:**
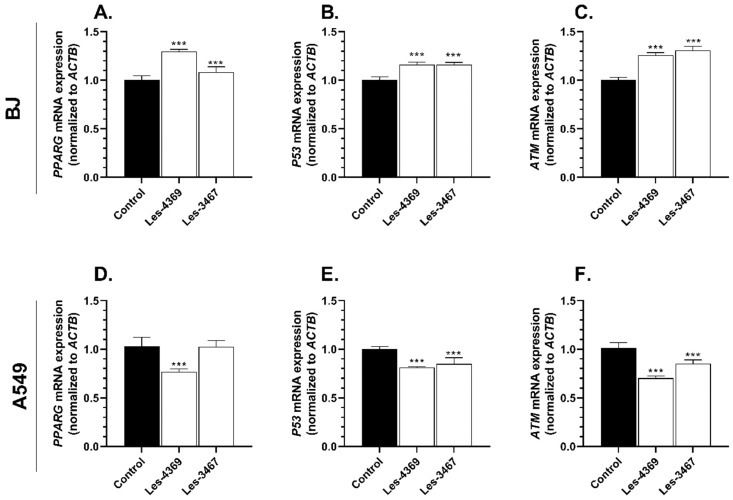
Effect of 1 μM Les-3467 and 1 μM Les-4369 on *PPARG*, *P53*, and *ATM* mRNA expression in the BJ normal cell line (**A**–**C**) and the A549 cancer cell line (**D**–**F**) after 24 h treatment with the tested compounds. The statistical significance of each data point was analyzed by Tukey’s test using one-way ANOVA for each study group; *** *p* < 0.001, compared with control cells.

After the 24 h exposure, the mRNA expression in the BJ and A549 cells was examined for the concentration of 1 μM of Les-3467 or 1 µM Les-4369. In the BJ cells, Les-4369 induced a significant 28.72% increase in the *PPARG* mRNA expression, while the Les-3467 compound increased the *PPARG* mRNA expression by 7.70%, compared to the control (Figure 9A). The *P53* mRNA expression increased significantly by 15.59% in the Les-4369 variant and by 15.60% in the Les-3467 treatment, compared to the control (Figure 9B). In the case of the *ATM* mRNA expression, Les-4369 increased this parameter by 25.50%, compared to the control. The Les-3467 compound increased the *ATM* mRNA expression by 30.57%, compared to the control (Figure 9C).

In the A549 cells, Les-4369 decreased the *PPARG* mRNA expression by 19.04%, compared to the control (Figure 9D). Les-3467 did not significantly change the *PPARG* mRNA expression. In turn, Les-4369 and Les-3467 decreased the *P53* mRNA expression by 19.04 and 15.43%, respectively, compared to the control (Figure 9E). Both Les-4369 and Les-3467 decreased *ATM* mRNA by 30.58 and 15.94%, respectively, compared to the control (Figure 9F).

**Figure 10:**
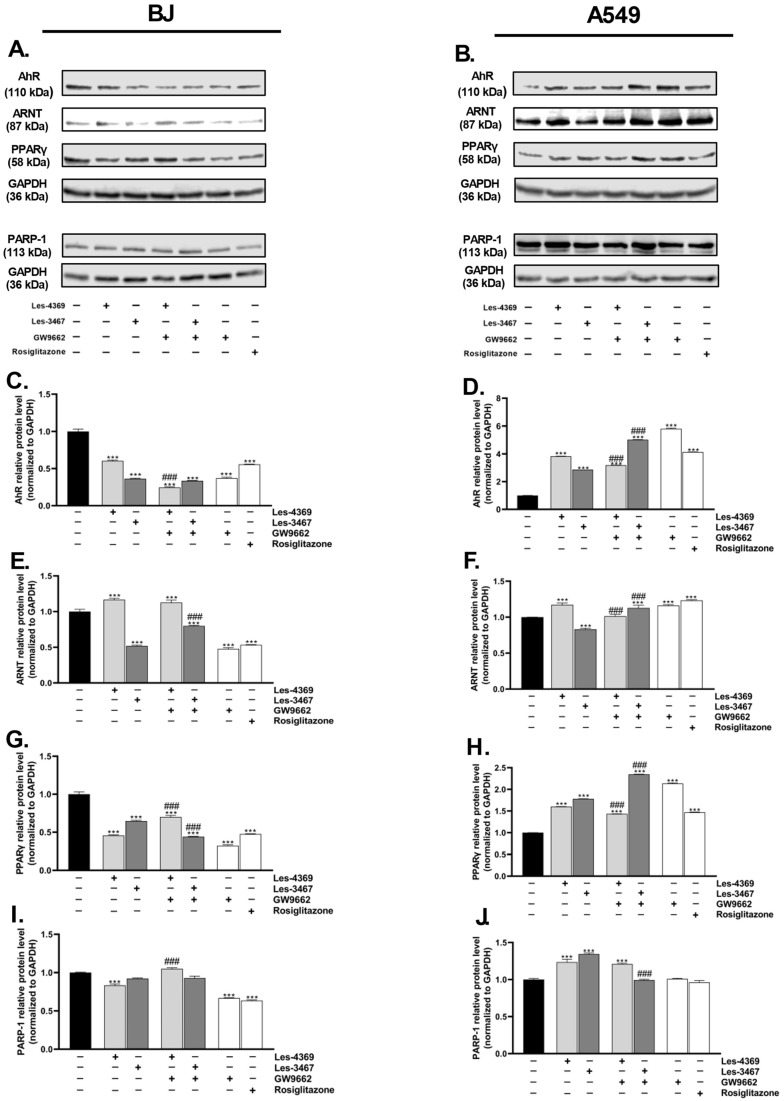
Representative Western blot of AhR, ARNT, PPARγ, and PARP-1 protein levels from BJ (**A**) and A549 (**B**) cells treated with 1 µM Les-4369 and Les-3467, 1 µM GW9662, 1 µM rosiglitazone, and co-treatment with Les-4369 and GW9662 or Les-3467 and GW9662. Western blotting bands for levels of AhR (**C**,**D**), ARNT (**E**,**F**), PPARγ (**G**,**H**), and PARP-1 (**I**,**J**) protein expression in BJ after 24 h treatment. The blots were stripped and reprobed with anti-GAPDH antibodies to control the amounts of protein loaded onto the gel. The statistical significance of each data point was analyzed by Tukey’s test using one-way ANOVA for each study group; *** *p* < 0.001 versus the control group; ### *p* < 0.001 versus the GW9662-stimulated group.

After the 24 h exposure of the BJ cells to Les-4369, Les-3467, co-treatment with Les-4349 and GW9662, co-treatment with Les-3467 and GW9662, treatment with GW9662 (PPARγ antagonist) alone, and rosiglitazone (PPARγ agonist), the AhR protein expression decreased by 39.55, 63.61, 75.47, 66.39, 62.90, and 44,31%, respectively, compared to the control (Figure 10C). In the case of the cancer cell line A549, the AhR protein expression was increased by Les-4369, Les-3467, co-treatment with Les-4369 and GW9662, co-treatment with Les-3467 and GW9662, GW9662, and rosiglitazone by 283.06, 186.81, 218.34, 402.65, 482.06, and 313.99%, respectively, compared to the control (Figure 10D). In the BJ cells, the ARNT protein expression was decreased in the groups treated with Les-3467, co-treatment with Les-3467 and GW9662, GW9662, and rosiglitazone by 48.00, 20.03, 52.16, and 46.66%, respectively, compared to the control (Figure 10E). In turn, Les-4369 and Les-4369 with GW9662 increased the ARNT protein expression by 16.65 and 12.68%, respectively, compared to the control (Figure 10E). In the A549 cells, the ARNT protein expression was increased in the variants with Les-4369, co-treatment with Les-4369 and GW9662, co-treatment with Les-3467 and GW9662, GW9662, and rosiglitazone by 17.10, 1.36, 12.87, 16.09, and 23.32%, respectively, compared to the control. However, Les-3467 alone decreased the protein expression by 16.81%, compared to control (Figure 10F). In the case of the PPARγ protein expression in the BJ cells, Les-4369, Les-3467, co-treatment with Les-4369 and GW9662, co-treatment with Les-3467 and GW9662, GW9662, and rosiglitazone decreased this parameter by 54.30, 35.36, 29.95, 55.88, 67.75, and 52,42%, respectively, compared to the control (Figure 10G). In turn, in the A549 cell line, the aforementioned experimental treatments increased the PPARγ protein expression by 60.14, 78.33, 43.43, 134.77, 113.41, and 47.08%, respectively, compared to the control (Figure 10H). The PARP-1 protein expression in BJ was decreased by 16.64, 33.17, and 36.41% in groups treated with Les-4369, GW9662, and rosiglitazone, respectively, compared to the control (Figure 10I). The protein expression in the A549 cells was increased by Les-4369, Les-3467, and co-treatment with Les-4369 and GW9662 by 23.54, 34.47, and 20.92%, respectively, compared to the control (Figure 10J).

Due to the increased caspase-3 activity induced by Les-3467 and Les-4369 in the BJ and A549 cells, we investigated the *ATM* mRNA expression, which may be involved in the induction of cell death via the response to genotoxic stress, as ATM is engaged in cell death as a result of DNA damage related to apoptosis [21]. Our results indicated higher *ATM* mRNA expression in the Les-3467 treatment than in the Les-4369 treatment. Interestingly, the results obtained for the A549 cells are the opposite to those for the BJ cells. The *P53* gene is also engaged in apoptosis, which is a result of cellular stress related to DNA damage [61]. For this reason, we also examined the *P53* mRNA expression, and we found increased mRNA expression induced by both compounds in the BJ cells. Les-4369 induced a higher decrease than Les-3467. However, both compounds decreased the mRNA expression in the A549 cells. Similar results for the *ATM* mRNA expression were obtained in the cancer line; Les-4369 caused a greater decrease in the mRNA expression, compared to Les-3467. The increased *P53* and *ATM* mRNA expression in the BJ cells can be interpreted as the development of cellular response to the applied Les-3467 and Les-4367. On the other hand, the decrease in the *P53* and *ATM* mRNA expression in the A549 cells can be interpreted as post-transcriptional inhibition of mRNA expression; this indicated a large amount of protein produced and rapid activation of related molecular pathways, which was confirmed at the protein level by the increase in the PARP-1 expression. However, we cannot exclude that the decrease in *P53* mRNA level after is the effect of the dysregulation of the *P53* molecular pathway in the A549 (cancer cells) line. Meanwhile, the increase in the *P53* mRNA expression in the BJ (normal cells) line could simply be the ability to regulate this pathway by the studied compounds. One of the anticancer drugs, doxorubicin (cytostatic), caused an increase in the *ATM* mRNA expression after 24 h in human skin cancer (G361), lung cancer (H460), breast cancer (MCF7), and colon cancer (HCT116) cell lines [62]. On the other hand, a potential anticancer compound, i.e., nerolidol (plant-derived), decreased the *ATM* mRNA expression in rat leiomyoma cells [63]. In the case of the *P53* gene, cisplatin (cytostatic) and the potential anticancer drug Veliparib (ABT888) increased the mRNA expression in the human tongue adenosquamous carcinoma cell line (CAL27) [64]. In turn, paclitaxel decreased the *P53* mRNA expression in breast cancer MDA-MB231 cells [65]. Cisplatin, Veliparib, and paclitaxel decreased the cell viability in investigated cell lines [64,65]. It is possible to suggest that the impact on the *ATM* and *P53* mRNA expression is related to the chemical structure or specificity of action against cancer cells. Moreover, the apoptosis induced by Les-3467 and Les-4369 may have also been caused by ROS production, as our results indicated that ROS production in the BJ and A549 cells was increased by both investigated compounds. Due to the increased ROS production, which is related with OS in cells, we also determined the *PPARG* mRNA expression, as PPARγ is known to regulate the expression of antioxidant enzymes [19,20]. The *PPARG* mRNA expression was decreased by Les-4369, but no changes were induced by Les-3467. The BJ cells exhibited an increase in the *PPARG* mRNA expression caused by Les-3467 and Les-4369. The changes in the *PPARG* mRNA expression in the BJ cells caused by both compounds and in the A549 cells exposed to Les-4369 may indicate an impact of Les-3467 and Les-4369 on the PPARγ pathway. Bar et al. (2022) described an increase in *PPARG* mRNA expression in BJ cells after 10 μM Les-3266 treatment [37]. Interestingly, Szychowski et al. (2022) demonstrated increased *PPARG* mRNA expression caused by 10 μM Les-236 in the same cell line and obtained similar results in the A549 cell line [53]. Another study reported decreased *PPARG* mRNA expression in SCC-15 cells treated with 10 μM Les-2194, Les-3377, and Les-3640 (Figure 4) [38]. TZDs can bind the PPARγ subtype and, in consequence, inhibit cancer cell proliferation, which indicates their anticancer properties [66]. An increase in the mRNA expression of these genes may suggest the involvement of 4-TZD derivatives in their activation in cell apoptosis. To confirm the involvement of the crucial proteins in DNA damage and OS in the BJ and A549 cells, we investigated the expression of AhR, ARNT, PPARγ, and PARP-1 proteins after the exposure to Les-3467, Les-4369, the PPARγ antagonist (GW9662), and the PPARγ agonist (rosiglitazone).

In the BJ cells, the AhR protein expression was decreased by all the studied compounds. GW9662 (PPARγ antagonist) potentiated the action of Les-4369. Interestingly, Les-4369 increased the ARNT protein expression, while Les-3467 decreased the expression of this protein, but GW9662 interfered only with Les-3467. Both compounds decreased the PPARγ expression, but, interestingly, GW9662 modified the Les-4369 and Les-3467 effects in different ways. Similarly, GW9662 prevented changes in the PARP-1 expression in the BJ cells after the exposure to Les-4369 and Les-3467. Interestingly, opposite effects of Les-4369 and Les-3467 on the AhR, ARNT, PPARγ, and PARP-1 protein expression were observed in the A549 cell line, compared to the BJ cells. Moreover, the results indicated that GW9662 (PPARγ antagonist) affected Les-3467 and showed synergistic action in the A549 cells. On the other hand, the impact of Les-4369 on the studied proteins in the A549 cells appeared to be independent of the GW9662 action and similar to rosiglitazone (PPARγ agonist). Therefore, we can summarize that Les-4369 and Les-3467 are at least PPARγ modulators. Moreover, the unclear protein changes, compared to those caused by GW9662 and rosiglitazone, may be explained by the well-described crosstalk between the AhR and PPARγ pathways. To date, it has been described that protein and mRNA expression is not always correlated. Moreover, there is a time shift and feedback mechanism between protein and RNA expression [67], according to which increased protein expression is accompanied by decreased mRNA expression and vice versa. Therefore, our observations of the PPARγ mRNA and protein expression are consistent with the current state of knowledge. This was particularly evident in our study, where the *PPARG* mRNA expression in the BJ cells increased upon the treatment with Les-3467 and Les-4367, whereas the PPARγ protein expression decreased upon the application of these compounds. Analogously, the *PPARG* mRNA expression in the A549 cell line was reduced by the Les-4369 treatment but did not exhibit significant changes in the Les-3467 variant. In turn, both compounds caused an increase in the PPARγ protein expression. Previously, Szychowski et al. (2023) found that both the PPARγ agonist (rosiglitazone) and the antagonist (GW9662) decreased AhR protein expression in mouse normal primary astrocytes [32], which is similar to our results for the BJ cells. Moreover, the VGVAPG (Val-Gly-Val-Ala-Pro-Gly) peptide (suspected to be a PPARγ modulator) enhanced the effect of GW9662 on AhR expression [32]. Gu et al. (2013) reported decreased ARNT protein expression in PPARγ-agonist-sensitive acute myeloid leukemia (AML) cells, whereas the ARNT protein expression in resistant AML was increased [68]. Therefore, these data may confirm our theory about the role of Les-3467 as a potential PPARγ agonist. To date, it has been described that Les-2194, Les-3166, and Les-6166 influence AhR protein expression in the 3T3-L1 cell line, which confirms that the new 4-TZD derivatives activate the aforementioned molecular pathway [69]. The cleavage of PARP-1 is initiated as a result of damage to DNA and by caspase-3 and -7 activity, which finally results in cell apoptosis [24,25]. Moreover, due to the crucial role of PARP-1 in the DNA repair system, the inhibition of PARP-1 leads to cell apoptosis as a result of damage to DNA, which creates a new strategy in the development of cancer drugs [70]. It has been described that both rosiglitazone and GW9662 do not significantly change PARP expression [71,72], which is similar to our observation for the A549 cells. On the other hand, Girnun et al. (2007) showed that rosiglitazone and carboplatin (cytostatic drug) cleaved PARP-1, while 0.5 μM rosiglitazone alone did not change PARP-1 expression and activation in A549 cells [73]. Moreover, the PPARγ agonist induced PARP-1 cleavage in breast cancer cell lines (MCF-7 and MDA-MB-231) [74]. Finiuk et al. (2017) described increased PARP-1 protein expression induced by the 4-TZD derivative Les-3833 (Figure 4) in human melanoma WM793 cells [6]. Another investigation conducted by Joshi et al. (2020) found increased amounts of cleaved (activated) PARP-1 in chronic myelogenous leukemic cells (K562) exposed to a 4-TZD derivative (Compound “3t”, Figure 4) [75]. Our results indicate an increase in the PARP-1 protein expression induced by the application of Les-3467 and Les-4369 alone, which may be a result of the high reactivity of these compounds. Therefore, Les-3467 and Les-4369 can potentially be PPARγ modulators and increase PARP-1 expression through this receptor. However, we cannot exclude direct DNA damage in the A549 caused by Les-3467 and Les-4369. As reported by Szychowski et al. (2019), 1 μM of the 4-TZD derivative Les-236 (Figure 4) slightly decreased PPARγ protein expression in BJ cells, and the effect of Les-236 was enhanced in co-treatment with GW9662 or rosiglitazone [53]. Moreover, the PPARγ agonist (rosiglitazone) increased PPARγ protein expression in the CACO-2 cell line, and the PPARγ antagonist (GW9662) increased protein expression in the SCC-15 cell line [53], which is similar to our results obtained for the A549 cells. In turn, other 4-TZD derivatives, i.e., Les-3377 and Les-3640 (2 μM), induced increased PPARγ protein expression in the 3T3-L1 cell line [45]. Szychowski et al. (2021) reported increased PPARγ protein expression caused by 10 μM Les-3377 and Les-3640 in SCC-15 cells [13]. The opposite results exhibited by normal cell lines and cancer cell lines may indicate different mechanisms of action of individual 4-TZD derivatives.

## 4. Conclusions

Our experimental results indicate cytotoxicity effects of Les-4369 and Les-3467 on BJ and A549 cells only at high µM concentrations. The mRNA expression results indicate the involvement of Les-4369 and Les-3467 in the mRNA expression of genes such as *PPARG*, *P53*, and *ATM*. In addition, Les-4369 and Les-3467 had an impact on the AhR, ARNT, PPARγ, and PARP-1 protein expression, which decreased in the BJ cells and increased in the A549 cell line. The present results may suggest weak anticancer properties of Les-3467 and Les-4369 due to their effects at high concentrations and non-selective action against BJ and A549 cells. However, we can summarize that Les-4369 and Les-3467 are at least PPARγ modulators.

## Data Availability

Dataset available on request from the authors.

## Abbreviations

4-TZD—4-thiazolidinone; ACTB—β-actin; AhR—aryl hydrocarbon receptor; ARNT—aryl hydrocarbon receptor nuclear translocator; ATM—ATM serine/threonine kinase; BCA—bicinchoninic acid; BSA—bovine serum albumin; CAD—caspase-activated DNase; CAT—catalase; CHAPS—3-((3-cholamidopropyl) dimethylammonio)-1-propanesulfonate; DCF—dichlorodihydrofluorescein; DMEM—Dulbecco’s Modified Eagle’s Medium; DMSO—dimethyl sulfoxide; DTT—dithiothreitol; ECL—chemiluminescent substrate; EDTA—ethylenediaminetetraacetic acid; ER—endoplasmic reticulum; F12K—Ham’s F-12K (Kaighn’s) medium; FBS—fetal bovine serum; GAPDH—glyceraldehyde 3-phosphate dehydrogenase; GSTA2—glutathione S-transferase A2; H2DCFDA—2′,7′-dichlorodihydrofluorescein diacetate; H33342—bisbenzimide trihydrochloride; HEPES—4-(2-hydroxyethyl)-1-piperazineethanesulfonic acid; ICAD—inhibitor of caspase-activated DNase; INT—iodonitrotetrazolium chloride; JSP-1—JNK simulating phosphatase-1; LDH—lactate dehydrogenase; MPMS—methoxyphenazine methosulfate; NAD—beta-nicotinotinamide adenine dinucleotide sodium salt; NADPH—nicotinamide adenine dinucleotide phosphate; NQO1—NAD(P)H quinone dehydrogenase; OS—oxidation stress; P53—tumor protein 53; PARP-1—poly [ADP-ribose] polymerase 1; PBS—phosphate-buffered saline; PPARγ—peroxisome proliferator receptor gamma; RIPA—radioimmunoprecipitation buffer; ROS—reactive oxygen species; RT—reverse transcription reaction; SOD—superoxide dismutase; TNFα—tumor necrosis factor α; UGT1A1—UDP-glucuronosyltransferase 1A1; UGT1A6—UDP-glucuronosyltransferase 1-6; XREs—xenobiotic-responsive elements.
